# Correlation between Employee Performance, Well-Being, Job Satisfaction, and Life Satisfaction in Sedentary Jobs in Slovenian Enterprises

**DOI:** 10.3390/ijerph191610427

**Published:** 2022-08-21

**Authors:** Zinka Kosec, Stella Sekulic, Susan Wilson-Gahan, Katja Rostohar, Matej Tusak, Marta Bon

**Affiliations:** 1 Faculty of Sport, University of Ljubljana, 1000 Ljubljana, Slovenia; 2 Dental Division, Faculty of Medicine, University of Ljubljana, 1000 Ljubljana, Slovenia; 3 National Institute for Public Health, 1000 Ljubljana, Slovenia; 4 Faculty of Business, Education, Law and Arts, University of Southern Queensland, Springfield Central 4300, Australia

**Keywords:** work performance, job satisfaction, life satisfaction, sedentary employment, well-being

## Abstract

The purpose of this study was to explore the relationship between employees’ work performance and their well-being, job satisfaction, and life satisfaction in sedentary jobs in Slovenian enterprises using a mixed-methods research design. The quantitative component of the research included the responses to four selected questionnaires of 120 employees in 22 identified enterprises (out of 81), with more than 20 employees, having more than 85 percent sedentary jobs. Each of four questionnaires was chosen to cover one area of enquiry under the research foci of work performance, job satisfaction, life satisfaction and well-being. The statistical program STATA was used for data analyses. The analysis shows statistically significant positive correlations between employee performance and job satisfaction (r = 0.35), employee performance and life satisfaction (r = 0.28), life satisfaction and well-being (r = 0.33), and job satisfaction and well-being, whereas the correlation between well-being and work performance did not prove to be statistically significant. The qualitative component of the mixed-methods research design included systematic observation combined with one-to-one discussions. The results indicated that job satisfaction and life satisfaction are more significant in determining work performance in sedentary jobs than employee well-being and that being unwell is still considered a sign of weakness; therefore, employees who are unwell do not want to expose themselves and refuse to cooperate in activities and studies about well-being. Further research examining the impact on work performance of organizational climate measurements in sedentary jobs is recommended.

## 1. Introduction

A person’s patterns of thinking and feelings are affected by internal and external environments in their life, including their profession and work conditions as some of the most important factors [1], which in turn have a negative impact on their lifestyle and work performance. Employers should be aware of the many factors that influence work environment, job and life satisfaction, well-being, and mental health, especially in sedentary jobs, since sedentary behavior has become a significant health issue in a post-industrialized world [1,2,3] and part of the dissatisfying lifestyle of many employees. Workplace environments are target settings for introducing processes of intervention to reduce sedentary behavior [1,2,3,4]. Different approaches designed to implement employees’ greater range of motion and standing during work hours have come to the fore [5,6,7,8,9,10]. Standing desks or desks that can accommodate standing or sitting have been introduced into work environments. Many companies provide different programs and equipment for their employees, active breaks during work hours, and policies about taking a break from the screen [3], which is especially recommended for older employees [5,6,7,8,9,10]. There is a lot of evidence that sedentary behavior influences the quality of life [1,2,3,4,5,6,7,8,9,10] and productivity [11]. Several studies have found that prolonged sitting time leads to cognitive impairment [10], mobility limitation [8], increased risk of mortality [12], and reduced quality of life in general [5,6,7,8,9,10,11,12].

Many companies have been trying to gain a sustainable competitive advantage by improving the effectiveness of work engagement interventions [13]. Work engagement, i.e., work performance, refers to a positive, fulfilling, work-related state of mind that is characterized by vigor, dedication, and absorption [14]. Work performance is defined as the total expected value to the organization of discrete behavioral episodes that an individual carries out over a standard period [15].

Organizations that focus on their employees’ welfare believe that employees’ attitudes and behaviors play a key role in improving the performance of an organization [13,16]. The organizational climate reflects employees’ perceptions of the policies, practices, and procedures that are expected, supported, and rewarded through the human resources department of the organization [17]. The organizational climate is a meaningful component with significant implications in human resource management and organizational behavior [16]. A complete reference guide, interventions, and policies to enhance employees’ well-being exist [17,18]. Environmentally sound behavior can be recognized through employees’ well-being and satisfaction, which are fundamental to employees’ quality work performance within organizations, particularly for employees in sedentary jobs, who often perform cognitive tasks that need a clear mind [19,20,21]. The effectiveness of physical activity interventions in improving well-being across office-based workplace settings [22], the association of sedentary behavior with metabolic syndrome [23], as well as the relation between financial incentives, motivation, and performance [24], are issues that fueled a great deal of research in the fields of management, occupational health, work and organizational psychology [15,16,17,18,19,20].

Although there is no consensus about a single definition of well-being, there is a general agreement that well-being includes the presence of positive emotions and moods (e.g., contentment), the absence of negative emotions (e.g., depression and anxiety), satisfaction with life, fulfillment, and positive functioning [16,17,18,19,20,21,22]. Well-being has been defined as the combination of feeling good and functioning well; the experience of positive emotions such as happiness and contentment as well as the development of one’s potential, having some control over one’s life, having a sense of purpose, and experiencing positive relationships [17,18,19,20,21]. Researchers from several areas have examined diverse aspects of well-being [17], i.e., physical, economic, social, emotional, and psychological well-being, development and activity, life satisfaction, domain-specific satisfaction, engaging activities, and work [17,18].

Empirical studies report strong correlations between social contact as well as health and subjective well-being [19]. Research on employees’ well-being operating in organizations was only developed a few decades ago. The examination of the relationship between employees’ well-being and the cardiovascular system, for example, revealed that physical and psychological well-being should be understood as a source of effectiveness [12,19]. In the past two decades, considerable development in the economics of subjective well-being is reflected in the great number of research studies published reporting the quality of life and its determinants [14,15,18,21,22,24].

Subjective well-being is a concept generally operationalized as multifaceted in nature, with both affective and cognitive components [17,18,25].

Among the constituent components of subjective well-being, life satisfaction was identified as a distinct construct representing a cognitive and global evaluation of the quality of one’s life as a whole [17]. Although life satisfaction is correlated with affective components of subjective well-being, it forms a separate factor from the other types of well-being [18,25]. Comprehensive assessment of subjective well-being requires separate measures of both life satisfaction and affective components of subjective well-being [21].

Life satisfaction is a cognitive evaluation of the overall quality of one’s life [21] and is one of the many overlapping facets of subjective well-being [25]. Life satisfaction is related to self-perception [26] and is a significant predictor of employees’ productivity in sedentary jobs [11], specifically in older adults [6,7,8,9].

Various studies [27,28,29,30] analyzed factors associated with life satisfaction and well-being and investigated what makes people happy [31]. The effect of age and body composition of office employees was examined [32], as well as stress and resilience potential [33] in different professions [34]. In such studies, the authors mentioned methodological limitations relevant to measurement scales [35], empirical models’ validations [36], statistical power analyses in behavioral science [37,38,39,40], and other principles and applications of qualitative research [41].

Life satisfaction judgments are mostly based on a person’s subjective criteria rather than necessarily reflecting outward conditions [25,26,29]. However, the assessment of life satisfaction can be only marginally influenced by mood and context since life satisfaction is a temporally stable construct [26]. Life satisfaction evaluations are broadly associated with other stable traits. The empirical relationships are consistent with the theory regarding core self-evaluations, which suggests that dispositions are important explanatory variables for predicting various forms of subjective well-being [17,18,19,22,27,28].

Job satisfaction is the result of a person’s attitude towards work and the factors associated with their work and life in general [15,16,21,22] and is closely related to work performance [15,16,21,22,31]. Several studies found a positive correlation between job satisfaction, the organizational climate [16], and overall performance [21,22].

Many authors mentioned other methodological dilemmas, i.e., different measurement scales [35] and empirical validations [36,40], i.e., also the calculation of posterior distributions by data augmentation [41], and different variations of satisfaction surveys [42]. Unfortunately, many studies on workplace characteristics, well-being, and life and job satisfaction rely primarily on cross-sectional self-reported surveys [8,26,27,28,29,43], making it difficult to disentangle the relationship between constructs. It has been a trend lately to develop work environment by various systematic approaches, e.g., the Human Resources Index [HRI] measurement [43]. In addition, motivation, and more specifically intrinsic motivation, was an important determinant of psychological well-being, gaining greater influence among male participants who had a higher level of physical activity, highlighting the need to increase one’s intrinsic motivation [44]. There are also always questions connected to lifestyle, in modern society especially related to eating habits [45]. The dynamic, adaptable complex approaches are especially important in recent years in response to COVID-19, connected with changes in general lifestyle, physical activity patterns, and sedentary behavior and associations with mental health [44,46,47,48,49], especially in computer workers, as one of the most typical sedentary works. In recent years, authors have suggested different models for the balance between work and life for subjective well-being, e.g., the moderated mediation model [50], or they have written about exploring the nature and antecedents of employee energetic well-being at work and job performance [51]. A special case is also well-being at work after a return to work [52]. This was considered as not under the special focus of our research; however, it was recognized as part of the organizational culture in the enterprises.

The purpose of this study was to explore the relationship between employees’ work performance and their well-being, job satisfaction, and life satisfaction in sedentary jobs in Slovenian enterprises with more than 80% sedentary workplaces, using a mixed-methods research design. This is the first time that research has been conducted into the correlation between employee performance, well-being, job satisfaction and life satisfaction in Slovenian enterprises, making the research a unique contribution to the field. The main gaps, which are supplemented by our studies, encourage similar further studies in sedentary jobs in Slovenia with the final goal to improve not only work performance but also the organizational culture in enterprises with sedentary jobs in Slovenia.

## 2. Materials and Methods

Both quantitative and qualitative methods were applied. All authors collaborated to design the procedure, while the first author carried out data collection. The possibility of a face-to-face or telephone conversation to explain further details of this study was offered to all participants and eleven of them used the opportunity to be provided with further information, while the remaining participants provided their consent to participate without asking for further explanation.

The methodological tool of this study was questionnaires, which have been used and proven in similar studies [15,25,36,38,42]. In addition, selected human resource management (HRM) professionals reviewed the questions to test the acceptance and feasibility of the questionnaire for our sample. To pilot test the questionnaire prior to the beginning of the trial, HRM professionals were approached that had been identified as being willing to volunteer to use the questionnaire. The data sets were analyzed quantitatively using descriptive statistics and analysis of reliability (STATA).

### 2.1. Quantitative Methodology

The first part consisted of a set of broad, self-report, psychometrically valid questionnaires conducted by the first author in the 22 organizations that have mostly (more than 90%) sedentary workplaces in Slovenia. A short explanation of the basic terminology used was added as an introduction to the questionnaires relating to work performance, well-being, job satisfaction, and life satisfaction.

### 2.2. Study Participants and Data Collection

The research team initially sent invitations with an explanation of the purpose of this study to the 81 identified enterprises, spending more than 85% of working time in sedentary positions. After detailed explanations, 22 of the invitees agreed to cooperate. Permissions and guidelines for the testing protocols and the design of this study, as well as any additional information required, were established through several face-to-face meetings and telephone conversations with executive managements and HRM specialists of the selected enterprises participating. In the pre-phase, the participant–employees were also offered the possibility of a face-to-face or telephone conversation about any details or additional information they required about this study. Eleven employees asked for additional information. Data collection was carried out from September 2018 to April 2019, with one day spent in each enterprise. Completion of all measurements for this study took approximately two hours per participant, between 9:00 A.M. and 3:00 P.M. To ensure standardized conditions, data collection took place in a designated meeting room which was intimate while also being large enough for completing all required measurements. Employees were from different levels of the organizational hierarchies and were categorized according to their role, gender, age, and education level (Table 1). Each employee was required to work an eight-hour day, starting between 6:00 A.M. and 9:00 A.M. and finishing between 2:00 P.M. and 5:00 P.M. (Table 1).

### 2.3. Procedure

All authors collaborated to develop the design of the procedure, while data collection was carried out by the first author.

Study participants were informed in advance of the purpose of this study, guaranteed anonymity and that the data analysis would be based on the responses of all organizations as a whole and not at the individual company level.

In the first phase of the procedure, conversations with employees who wanted further explanation were carried out. The questions referred to the aims of this study, the topics, the hypothesis, if any, as well as the conducted research and their results. The remaining participants provided consent to cooperate without asking for further explanation. After a positive response from all the participants, the testing procedure was carried out in the participants’ workplace. A short explanation of basic terminology used was also added as an introduction to the questionnaire.

The aim of this study was to collect information about four components of work: (i) employee performance; (ii) well-being; (iii) job satisfaction; and (iv) life satisfaction. The first part consisted of a set of broad, self-report, psychometrically valid questionnaires. The adapted self-assessment questionnaires were validated and translated into Slovenian.

The following self-reported questionnaires were used; one for each of the four components of work being researched. That is, employee performance, well-being, job satisfaction, and life satisfaction.

- Employee performance: The Employee Performance Questionnaire (EPQ) [38] (Capital Associated Industries, Inc. (Raleigh, NC, USA), 2011) is a valid [36] measure that assesses individuals on different parameters related to a wide range of working skills (e.g., working at full potential, quality of work, consistency of work, communication, independence, taking initiative, teamwork, productivity, creativity, honesty, integrity, relationships with colleagues, relationships with customers, technical knowledge, reliability, accuracy, and presence). It consists of 23 items with one reverse question and five response options: One participant indicated that the suggested questions did not apply to them, while five participants indicated aptitude. The EPQ is characterized by a total score with a possible range of scores from 23 to 115.
- Well-being: The General Health Questionnaire (GHQ) [42] is a consistent, reliable self-report questionnaire designed for use in a variety of settings and cultures in general population samples. There are several versions of the GHQ [42]. In this study, we used the GHQ-12 due to the simplicity of application in practice and research. The selected version consists of 12 items that examine the mental health of individuals by rating a specific symptom experience or current behavior on a 4-point scale (less than usual, no more than usual, rather more than usual, or much more than usual). It is characterized by a total score of 12–36.
- Job satisfaction: The Job Satisfaction Questionnaire (JSQ) [42] is a psychometrically valid self-report questionnaire that measures an individual’s job satisfaction [42]. It consists of 13 questions and five response options, with 1 indicating strong disagreement and 5 indicating strong agreement with the suggested statements. It is characterized by a total score in the range of 13–65.
- Life satisfaction: The Life Satisfaction Questionnaire (LSQ) [15,25] is a brief psychometrically based 5-item instrument designed to measure global cognitive assessments of life satisfaction. It consists of five items and seven response options, from 1 indicating strong disagreement to 7 indicating strong agreement. The LSQ has excellent psychometric properties, including high internal consistency and test–retest reliability. It is characterized by a total score in the range of 7–35.

The data collected from the questionnaires were accompanied by systematic observation, which was introduced as an objective, well-ordered method for close examination of the selected aspects of this study. Systematic observation involved questions about the participants’ opinions on concrete activities to promote health and well-being in the organizations, on life and job satisfaction in sedentary jobs, and on why some employees decided to cooperate and some not. Systematic observation and a number of in-person, one-to-one discussions were undertaken in the same session of the preparation phase, especially with people who supported the authors in organizing data collection in the company (mostly HR specialists or directors), and later with the respondents while conducting the survey.

The Ethical Committee at the Faculty of Sports, the University of Ljubljana (No. 5) approved this study in March 2018.

### 2.4. Data Analyses

The statistical software STATA (Stata Statistical Software: Release 14.2, rev.19; 2016, StataCorp LP, College Station, TX, USA) was used to analyze sample data.

Using descriptive methods, the sample was analyzed by taking measurements of the frequency and percentages of responses to all questions. The statistical analysis was blinded to the researchers and conducted independently. Descriptive statistics, such as proportions for categorical variables and mean values and standard deviations for numeric variables, were used to summarize respondents’ characteristics.

Two-Sample Assuming Equal Variances (*p* = 0.05) was used to calculate the differences between groups according to:

- Age (range 19–35; age range 36–70),
- Gender (man/woman),
- BMI (normal weight = 18.5–24.9; pre-obesity = 25.0–29.9), and
- Education level (high school degree = 2; college and university degree = 3).

Respondents’ self-report EPQ, GHQ, JSQ, and LSQ scores were summarized with an average score for each question (for each individual). The correlation between the results of the self-assessed variables from the questionnaires (the EPQ, the GHQ, the JSQ and the LSQ) was applied, where the magnitude of correlation coefficients was explained according to Hemphill [39]. The effect size was considered as low when the value ranged from 0.1 to 0.3, moderate when it ranged from 0.3 to 0.5, and large when it ranged from 0.5 to 1.0 [41]. Multiple regression analysis was used to assess the relationship between one dependent variable calculation (the EPQ, which consisted of 23 variables), and three independent variables (the GHQ consisting of 12 items, the JSQ of 13, and the LSQ of five items). R-squared (R^2^) was used to measure a proportion of explained variance represents the fit of the data to the model. The effect size was considered low when R^2^ was <0.3, no effect or very weak when R^2^ was 0.3, medium when R^2^ was 0.5, and large when R^2^ was 0.7 [41].

Adjusted R-squared measures were used to test the fit of the model.

### 2.5. Qualitative Methods

The qualitative research methodology was mostly followed according to Evans et al. [41].

Question-focused analysis was used as a starting point when organizing the raw data, and the responses that had similar themes and that represented the same points were grouped together. All the information was transcribed verbatim and read through several times by the authors. The first-named author then conducted a thematic analysis according to Braun and Clark and Evans et al. [41], whereby initial comments, codes and memos were categorized systematically into broader themes and concise phases as evident in Table 2. The six phases identified were (i) becoming familiar with the data, (ii) generating initial codes, (iii) identifying potential themes, (iv) reviewing themes, (v) defining and naming the themes and (vi) producing the report.

The qualitative method involved information about specification of the exact actions, attributes, and other variables that were systematically written in the preparation phase and after each data collection, through administration of questionnaires in all organizations. With this observation, the authors aimed to explore how decisions were made and provided the researchers with detailed insight. The data analysis followed the principles of qualitative methodologies [41].

The main questions in the one-to-one discussion were:

- What is the reason that you agree to participate in actives connected with work performance, job satisfaction and life satisfaction measurements (also in this study)?
- What is your opinion about the significance of job satisfaction, life satisfaction and well-being measurements for work performance?
- What is your opinion about employees’ willingness/unwillingness to participate in actives connected with work performance and your opinion about the general organizational climate in the enterprises?
- Should companies in Slovenia invest more in employees’ work performance (in their well-being, job, and life satisfaction)? If yes/no, what are your reasons?

## 3. Results

### 3.1. Demographic Data of the Participants

A convenience sample of 120 employees from 22 organizations—65 of whom were female, with an age range from 25 to 69 years, and 55 of whom were male, with and age range from 22 to 70—participated in this study. The main criterion was having a sedentary job. Employees were of different levels of the organizational hierarchies: operational workers (57%), management (9.8%), division management (9.1%), directors and owners (3.3%), and sole traders (14.0%). The study participants were also categorized according to their education level (Table 1).

A total of 120 respondents from 22 organizations completed the EPQ, the GHQ, the JSQ, and the LSQ (Table 1).

The mean age of the participants (SD) was 35.1 (±12.9) years and more than half of them were female (53.3%). The mean height and weight of the participants were 1.7 m and 74.3 kg, respectively, which was considered ‘normal weight’ when assessing the body mass index (BMI) of the participants according to the World Health Organization BMI classification [45].

Among the organizations, 39.3% of all employees worked in a small organization with the working group of less than 10 employees, which is the highest proportion in the sample; 20.5% worked in a group of 11–50 employees; 28.7% in a group of 51–250 employees; only 11.5% of all employees worked in a group with more than 250 employees.

The majority of study participants (41.0%) had a secondary school diploma or bachelor’s degree prior to the Bologna Process, while 38.5% had completed secondary schooling and 16.4% a master’s or specialization or Ph.D.

EPQ: The EPQ was measured on a on a scale of 1–5. Employees assessed their own work performance as high; the mean score of the EPQ reached 4.2 (SD = 0.04), which is a high score. Accordingly, the differences between the respondents were minor. The lowest value was 3.1, and approximately 80% of the estimates were higher than 4.0.

GHQ: The mean value of the GHQ on a scale of 0–3 was 1.38 (SD = 0.04). The scores were almost symmetrically distributed. The differences between respondents were typical of normal distribution.

JSQ: The JSQ was measured on a scale of 1–5. The mean value of the JSQ was 3.84 (SD = 0.06). Similarly to the EPQ, the JSQ scores showed progress in a positive direction and little difference between respondents. The lowest score was 0.17, while the highest score was 2.75.

LSQ: The LSQ scores were measured on a scale of 1–7, where the mean value was 4.86 (SD = 0.11). The differences between respondents were significant. The lowest mean value was 1.67, and the highest was 7.0. Nearly ten percent (9.8%) of the respondents reported dissatisfaction with work, with a mean value of <3. More than 80% of respondents reported their satisfaction with work, with a score of four or more.

### 3.2. Employee Work Performance and the Selected Variables (Well-Being, Job and Life Satisfaction)

The correlations between the Employee Performance Questionnaire (EPQ) and the selected factors from the GHQ (well-being), by the JSQ (job satisfaction) and by the LSQ (life satisfaction) were measured with correlation and regression analysis.

The analyses of the results showed statistically significant positive correlations between estimates of the EPQ and the JSQ (r = 0.36) and between estimates of employee performance and life satisfaction (r = 0.29). Cohen’s effect size was medium, showing no correlation between employee performance and general health (r = −0.08), possibly a negative correlation between the two measures although not statistically significant (*p* = 0.33) (Table 3).

Multiple linear regression was calculated to predict work performance based on the GHQ, JSQ and LSQ results. A significant regression equation was identified, F (3, 116) = 7.70, *p* = 0.0001, with an R^2^ of 0.166.

Participants’ EPQ result was equal to 3.109 ± 0.066; GHQ 3.109 ± 0.181; JSQ 3.109 ± 0.076; LSQ (with GHQ, JSQ, and LSQ scores measured as means).

**Figure 1:**
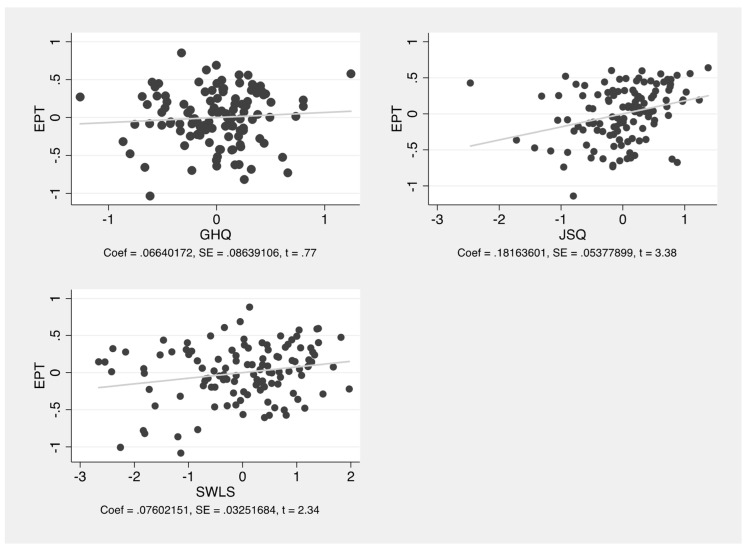
Scatter plots of the EPQ associated with the GHQ, JSQ, and satisfaction with life scale (SWLS = LSQ) means in the regression model. Coeff. (coefficient), SE (standard error), and t (t-statistic).

Both the JSQ (*p* = 0.001) and LSQ results (0.021) significantly affected the EPQ values, while the GHQ results (0.444) did not. A graphical representation of the correlation from the regression model is shown in detail in Figure 1.

### 3.3. Qualitative Method Results

Thematic analysis was used as a starting point after organizing the raw data, and the responses that had similar themes and that represented the same points were grouped together.

More than expected results and themes were found for the final report from thematic analysis:

Systematic observation

- Employees who explain their overall status as ‘healthy and wealthy’ and themselves as ‘a productive employee’ are ready to cooperate in research.
- Employees who are not in good health try to hide their condition and are not ready to speak about it in a company setting.
- Employees who are not in good health feel vulnerable and deny all sorts of activities in the enterprises.
- In the testing process, the study participants insisted that the data only be analyzed as part of the whole sample and not on an individual basis or within one company.
- Employees who were not ready to cooperate are also not ready to take part in other healthy lifestyle activities being organized in the frame of company.
- Employees who are not ready to take part in this research also in general refuse nearly all ‘well-being and social lifestyle’ activities in the enterprise and in their leisure time.

One-to-one discussions:

- The respondents (employees in the enterprises who were ready to take part in this research) reported that employees from all companies in general are divided into two groups concerning work performance topics—those willing to participate and those who would absolutely not. They were always on the opposite ends of the spectrum, which could mean that cohesion in not high and that the organizational climate is not optimal.
- Employees who were ready to participate reported their opinion that they represented the better part of employees in the organizations, that they always cooperate, that they are more motivated for better work performance and that they are more productive. They call themselves cooperative employees.
- The cooperative employees reported that there are some employees in the enterprises who are not cooperative, because they try to hide their level of well-being, their health and lifestyle status.
- According to management representatives, employees who are not in good health feel vulnerable and refuse to participate in all sorts of activities organized in their company.
- Respondents reported that employees who were not ready to take part in this research (called ‘those others’) also in general refuse to participate in nearly all well–being and social activities in their company and in their leisure time.
- Respondents reported that “those others” are not motivated and are not concerned with creating a good organizational climate.
- Although anonymity in the testing process was provided to all, the participants reported concerns and doubts, insisting that the data should only be analyzed as part of the whole sample and not on an individual basis or within one company.

Thematic analysis (coding and iterative comparison) gave some interesting conclusions (Table 4).

## 4. Discussion

The labor market is constantly changing, and sedentary work behavior is nowadays, due to technological advancement and new lifestyles, becoming even more pervasive worldwide. One of the questions is how the new conditions influence work performance, responsibilities, and ability to do the job well. This motivated our research on sedentary jobs for the first time in Slovenia together with well-being and other characteristics. The primary purpose of this study was to determine the correlation between work performance and different factors (well-being, job, and life satisfaction) in sedentary jobs. The results show statistically significant correlations between work performance and two measured factors—job satisfaction and life satisfaction. On the other hand, the correlation between well-being and work performance surprisingly did not prove to be statistically significant. Nevertheless, our results showed that well-being is significantly correlated with job and life satisfaction, which are correlated with work performance. On that basis, it can be concluded that there is some indirect relationship between work performance and well-being, which was also established in some earlier studies [19,23,26].

The correlations between job satisfaction [14,15], life satisfaction [5,6], and work performance have already been proven in many countries. It has also been found that sedentary behavior negatively correlates with an active lifestyle [4,6] and with less effective work performance [14,35], which also supports our conclusions. Furthermore, our systematic observation findings indicate specific problems in the organizational climate among employees and point to a significant division between the groups and consequential low team cohesiveness, which is essential for team or group effectiveness and work performance [50]. In our study, the group of employees who were willing to participate called themselves ‘cooperative employees’, whereas employees who were not ready to take part in this study were referred to as ‘those others’, those who never cooperate and always complain. We regret that we were not able to conduct one-to-one discussions with the ‘those others’ group and determine the reasons for their refusal to participate. Many respondents reported their opinion that those who refused to participate in this study in general create a negative working atmosphere in the studied companies. Such opinions were also confirmed by the opinion of management representatives. This calls for new approaches for improving the general organizational climate in Slovenian enterprises, as a base for other necessary improvements. Our findings could, therefore, also serve as an incentive to develop new practical interventions and approaches to improving the organizational climate, as the main goal is to improve work performance and thus all factors that might affect it.

Job satisfaction can be improved in practice by encouraging employees and making them encourage other employees [14,15,20,21,30], which also improves team cohesion [37], by giving them access to information and all necessary resources to perform their job efficiently, giving them real-time feedback on their job performance [43] and by providing them with opportunities to explore and show their skills and talents. Furthers studies are needed to confirm whether the employer’s trust and faith in their employees are crucial, a subject studied by others [21,30,44,46,47,48]. The participants, however, believe that the biggest hindrance to achieving such improvement are employees who are not ready to cooperate.

The findings from this study also led to the conclusion that sedentary jobs in the studied companies require complex human resource management. Therefore, more complex studies are needed in this field, with special monitoring and maybe even with human resource index (HRI) measurements, e.g., [43], which is the current trend in economics, as well as the new reality in economics [47,48,49,50,51,52,53,54] and in society.

## 5. Conclusions

As in most of Europe, Slovenia is also facing the challenge of sedentary behavior as part of modern work conditions. This is the first time that Slovenian enterprises were researched in terms of sedentary work conditions, concerning job satisfaction, life satisfaction and well-being on work performance, which is the main novelty of the work and presents the possibility of comparing findings with other studies [48,49,50,51,52,53,54], such as the effect of COVID-19 [5,47], remote job options and cross-country differences [53] or socio-economics status in the relationship between leadership and well-being [54]. The main gaps, which are supplemented by our studies, are, in addition to finding the correlations between some factors and work performance in sedentary jobs, encouraging similar further studies with the final goal of determine the factors that correlate most with job performance in sedentary work conditions. The aim was to highlight that the study found many employees do not cooperate. In general, our study confirms that for employees in sedentary jobs in Slovenia, work performance is correlated with life and job satisfaction. Nevertheless, it is not directly correlated with well-being as this may have been predicted based on the findings of previously published studies. This can be explained by the small sample size and data collection limitations due to distrusting the research, discomfort, or poor well-being in the work environment. This may suggest that the enterprises involved in our study are confident about their organizational climate. Our practical recommendation is to expand the focus from work performance to improving cohesion and the organizational climate in enterprises in order to create the optimal work environment in sedentary workplaces in Slovenia. The results indicate important conclusion as well as making clear the significant need for further research on the impact of well-being on employees’ productivity in sedentary jobs, in order to face the new reality requiring the need to organize sedentary jobs in different forms, e.g., providing remote job options which might be critical economically in this new decade.

## Figures and Tables

**Table 1:** General characterization of the participants.

Participants (N = 120)	N (%) or Mean (SD)
*M Age (years)*	35.1 (12.9)
*N Gender (female)*	64 (53.3)
**Physical characteristics**	
*Height (meter)*	1.7 (0.1)
*Weight (kilogram)*	74.3 (16.9)
*Body mass index*	24.4 (3.9)
**Groups of employees within organizations**	
*0–10*	47 (39.1)
*11–50* *51–250*	25 (20.8) 35 (29.1)
*≥250*	13 (10.8)
*Sedentary job (hours)*	7.65 (6.2)
**Education level**	
*Secondary school*	50 (41.6)
*Bachelor’s degree (also pre-Bologna)*	50 (41.6)
*Master’s degree and higher*	20 (16.6)
**Self-assessed questionnaires**	
*Employee performance (1–5)*	4.2 (0.3)
*General health (1–3)*	1.3 (0.4)
*Job satisfaction (1–5)*	3.8 (0.6)
*Satisfaction with Life (1–7)*	4.8 (1.1)

Note: N (number of participants); SD (standard deviation). Body mass index classification: underweight <18.4; normal weight 18.5–24.9; overweight 25.0–29.9; obesity ≥30.0.

**Table 2:** Estimated correlation matrix and the significance of self-report instruments.

Variables	Employee Performance	General Health	Job Satisfaction	Satisfaction with Life
**Employee Performance**	1.0000			
**General Health**	−0.0886 *0.3358*	1.0000		
**Job Satisfaction**	0.3557 * 0.0001	−0.2863 * 0.0015	1.0000	
**Satisfaction with Life**	0.2898 * 0.0013	−0.3277 * 0.0003	0.3135 * 0.0005	1.0000

Note: * Significance *p* < 0.05.

**Table 3:** Regression analysis between one dependent (EPQ) and three independent variables results (GHQ, JSQ, and LSQ).

	Regression Model
Variable	Coeff. (t)
Job Satisfaction	0.181 (3.38)
Satisfaction With Life	0.076 (2.34)
General Health	0.066 (0.77)
Constant	3.109 (10.54)
R-Squared (N)	0.166 (120)
Adj. R-Squared	0.144

Note: Coeff. (coefficient); t (t-statistic); N (number of participants). The standardized coefficient estimates the mean change in the dependent variable for a 1 standard deviation (SD) increase in the independent variable.

**Table 4:** Results of systematic observations and one-to-one dissuasions.

	Who	Life Satisfaction	Work Performance	Job Satisfaction	Final Themes
A	participants in this study	high	high	high	we ‘healthy and wealthy’
B	NOT READY TO COOPERATE				
C	executive management and HRM specialists	high	high	high	employees A are good; B have lower work performance
A about B	those others	not satisfied at all	low work performance	low	not in good health
					try to hide their level of well-being
					they are not productive
					bad work performance
					not good lifestyle
C about B		low	low	low	not in good health, they feel vulnerable; refuse to participate in all sorts of activities
C about A		high	high	high	they are our best employees; positive org. climate

## Data Availability

The data reported in this study are available on request from the corresponding author upon reasonable request. The data are not publicly available due to its proprietary nature.

## References

[1] Faller G. (2021). Future Challenges for Work-Related Health Promotion in Europe: A Data-Based Theoretical Reflection. Int. J. Environ. Res. Public Health.

[2] López-Valenciano A., Mayo X., Liguori G., Copeland R.J., Lamb M., Jimenez A. (2020). Changes in sedentary behaviour in European Union adults between 2002 and 2017. BMC Public Health.

[3] Munir F., Biddle S.J., Davies M.J., Dunstan D., Eslinger D., Gray L.J., Edwardson C.L. (2018). Stand More AT Work [SMArT Work]: Using the behaviour change wheel to develop an intervention to reduce sitting time in the workplace. BMC Public Health.

[4] Chu A.H., Ng S.H., Tan C.S., Win A.M., Koh D., Müller-Riemenschneider F. (2016). A systematic review and meta-analysis of workplace intervention strategies to reduce sedentary time in white-collar workers. Obes. Rev..

[5] Hwang J.-H. (2022). Mediating Effects of Psychological States on Work Performance of Visiting Nurses According to COVID-19 Workplace Quarantine Measures: A Multi-Group Path Analysis Study. Int. J. Environ. Res. Public Health.

[6] Choi J.I., Cho Y.H., Kim Y.J., Lee S.Y., Lee J.G., Yi Y.H., Tak Y.J., Hwang H.R., Lee S.H., Park E.J. (2021). The Relationship of Sitting Time and Physical Activity on the Quality of Life in Elderly People. Int. J. Environ. Res. Public Health.

[7] Meneguci J., Sasaki J.E., Santos A., Scatena L.M., Damião R. (2015). Sitting Time and Quality of Life in Older Adults: A Population-Based Study. J. Phys. Act. Health.

[8] Yen C.H., Ku M.H., Wang C.Y. (2017). Self-reported Sitting Time is Associated with Decreased Mobility in Older Adults. J. Geriatr. Phys. Ther..

[9] Lee Y.H., Kim H., Park Y. (2022). Development of a Conceptual Model of Occupational Stress for Athletic Directors in Sport Contexts. Int. J. Environ. Res. Public Health.

[10] García-Hermoso A., Ramírez-Vélez R., Celis-Morales C.A., Olloquequi J., Izquierdo M. (2018). Can physical activity attenuate the negative association between sitting time and cognitive function among older adults? A mediation analysis. Exp. Gerontol..

[11] Rosenkranz S.K., Mailey E.L., Umansky E., Rosenkranz R.R., Ablah E. (2020). Workplace Sedentary Behavior and Productivity: A Cross-Sectional Study. Int. J. Environ. Res. Public Health.

[12] Patterson R., McNamara E., Tainio M., de Sá T.H., Smith A.D., Sharp S.J., Edwards P., Woodcock J., Brage S., Wijndaele K. (2018). Sedentary behaviour, and risk of all-cause, cardiovascular and cancer mortality, and incident type 2 diabetes: A systematic review and dose-response meta-analysis. Eur. J. Epidemiol..

[13] Knight C., Patterson M., Dawson J. (2017). Building work engagement: A systematic review and meta-analysis investigating the effectiveness of work engagement interventions. J. Organ. Behav..

[14] Diener E., Seligman M.E. (2004). Beyond money: Toward an economy of well-being. Psychol. Sci. Public Interest.

[15] Spagnoli P., Haynes N.J., Kovalchuk L.S., Clark M.A., Buono C., Balducci C. (2020). Workload, Workaholism, and Job Performance: Uncovering Their Complex Relationship. Int. J. Environ. Res. Public Health.

[16] Bin Ahmad K.Z., Jasimuddin S.M., Kee W.L. (2018). Organisational climate and job satisfaction: Do employees’ personalities matter?. Manag. Decis..

[17] Ruggeri K., Garcia-Garzon E., Maguire Á., Matz S., Huppert F.A. (2020). Well-being is more than happiness and life satisfaction: A multidimensional analysis of 21 countries. Health Qual. Life Outcomes.

[18] Huppert F.A., Huppert F.A., Cooper C. (2014). The state of well-being science: Concept s, measures, interventions, and policies. Well-Being: A Complete Reference Guide, Interventions and Policies to Enhance Wellbeing.

[19] Wu T.-J., Wang L.-Y., Gao J.-Y., Wei A.-P. (2020). Social Support and Well-Being of Chinese Special Education Teachers—An Emotional Labor Perspective. Int. J. Environ. Res. Public Health.

[20] Clark B., Chatterjee K., Martin A., Davis A. (2020). How commuting affects subjective well-being. Transportation.

[21] Huang C., Xie X., Cheung S.P., Zhou Y., Ying G. (2021). Job Demands, Resources, and Burnout in Social Workers in China: Mediation Effect of Mindfulness. Int. J. Environ. Res. Public Health.

[22] Abdin S., Welch R.K., Byron-Daniel J., Meyrick J. (2018). The effectiveness of physical activity interventions in improving well-being across office-based workplace settings: A systematic review. Public Health.

[23] Edwardson C.L., Gorely T., Davies M.J., Gray L.J., Khunti K., Wilmot E.G., Biddle S.J. (2012). Association of sedentary behaviour with metabolic syndrome: A meta-analysis. PLoS ONE.

[24] Landry A.T., Gagné M., Forest J., Guerrero S., Séguin M., Papachristopoulos K., Information R. (2017). The relation between financial incentives, motivation, and performance. J. Personal. Psychol..

[25] Diener E., Lucas R.E., Oishi S. (2018). Advances and open questions in the science of subjective well-being. Collabra Psychol..

[26] Miller B.K., Zivnuska S., Kacmar K.M. (2019). Self-percEPQ ion and life satisfaction. Personal. Individ. Differ..

[27] Dolan P., Peasgood T., White M. (2008). Do we really know what makes us happy? A review of the economic literature on the factors associated with subjective well-being. J. Econ. Psychol..

[28] Donaldson S.I., Heshmati S., Lee J.Y., Donaldson S.I. (2020). Examining building blocks of well-being beyond perma and self-report bias. J. Posit. Psychol..

[29] Szcześniak M., Mazur P., Rodzeń W., Szpunar K. (2021). Influence of Life Satisfaction on Self-Esteem among Young Adults: The Mediating Role of Self-Presentation. Psychol. Res. Behav. Manag..

[30] Proctor C., Linley P.A., Maltby J., Levesque R. (2017). Life Satisfaction. Encyclopedia of Adolescence.

[31] Wijngaards I., Burger M., van Exel J. (2021). Unpacking the Quantifying and Qualifying Potential of Semi-Open Job Satisfaction Questions through Computer-Aided Sentiment Analysis. J. Well-Being Assess..

[32] Wick K., Faude O., Schwager S., Zahner L., Donath L. (2016). Deviation between self-reported and measured occupational physical activity levels in office employees: Effects of age and body composition. Int. Arch. Occup. Environ. Health.

[33] Today W.I., Dillon D. (2020). Community Gardening: Stress, Well-Being, and Resilience Potentials. Int. J. Environ. Res. Public Health.

[34] Hepburn S.-J., Carroll A., McCuaig-Holcroft L. (2021). A Complementary Intervention to Promote Wellbeing and Stress Management for Early Career Teachers. Int. J. Environ. Res. Public Health.

[35] Ackerman C.E., Warren M.A., Donaldson S.I. (2018). Scaling the heights of positive psychology: A systematic review of measurement scales. Int. J. Wellbeing.

[36] Rabindra P.K., Lalatendu Jena K.L. (2017). Employee Performance at Workplace: ConcEPQ dual Model and Empirical Validation. Bus. Perspect. Res..

[37] Salas E., Grossman R., Hughes A.M., Coultas C.W. (2015). Measuring Team Cohesion: Observations from the Science. Hum. Factors J. Hum. Factors Ergon. Soc..

[38] McLeod S.A. (2019). Likert Scale Definition, Examples and Analysis. Simply Psychology. https://www.simplypsychology.org/likert-scale.html.

[39] Hemphill J.F. (2003). Interpreting the magnitudes of correlation coefficients. Am. Psychol..

[40] Lucas R.E., Diener E., Suh E. (1996). Discriminant validity of well-being measures. J. Personal. Soc. Psychol..

[41] Evans A.B., Barker-Ruchti N., Blackwell J., Clay G., Dowling F., Frydendal S., Hybholt M.G., Hausken-Sutter S.E., Lenneis V., Malcolm D. (2021). Qualitative research in sports studies: Challenges, possibilities and the current state of play. Eur. J. Sport Soc..

[42] Smith S. (2020). Employee Satisfaction Surveys: 3 Sample Templates with Questions. www.qualtrics.com/blog/employee-satisfaction-survey.

[43] Molin F., Paulsson S.Å., Hellman T., Svartengren M. (2021). Can the Human Resources Index [HRI] Be Used as a Process Feedback Measurement in a Structured Support Model for Systematic Work Environment Management?. Int. J. Environ. Res. Public Health.

[44] Granero-Jiménez J., López-Rodríguez M.M., Dobarrio-Sanz I., Cortés-Rodríguez A.E. (2022). Influence of Physical Exercise on Psychological Well-Being of Young Adults: A Quantitative Study. Int. J. Environ. Res. Public Health.

[45] WHO Obesity and Overweight. https://www.who.int/news-room/fact-sheets/detail/obesity-and-overweight.

[46] Meyer J., McDowell C., Lansing J., Brower C., Smith L., Tully M., Herring M. (2020). Changes in Physical Activity and Sedentary Behaviour in Response to COVID-19 and Their Associations with Mental Health in 3052 US Adults. Int. J. Environ. Res. Public Health.

[47] Moreira S., Criado M.B., Ferreira M.S., Machado J., Gonçalves C., Mesquita C., Lopes S., Santos P.C. (2022). The Effects of COVID-19 Lockdown on the Perception of Physical Activity and on the Perception of Musculoskeletal Symptoms in Computer Workers: Comparative Longitudinal Study Design. Int. J. Environ. Res. Public Health.

[48] Teetzen F., Bürkner P.-C., Gregersen S., Vincent-Höper S. (2022). The Mediating Effects of Work Characteristics on the Relationship between Transformational Leadership and Employee Well-Being: A Meta-Analytic Investigation. Int. J. Environ. Res. Public Health.

[49] Thielmann B., Schnell J., Böckelmann I., Schumann H. (2022). Analysis of Work-Related Factors, Behavior, Well-Being Outcome, and Job Satisfaction of Workers of Emergency Medical Service: A Systematic Review. Int. J. Environ. Res. Public Health.

[50] Hasan Z.U., Khan M.I., Butt T.H., Abid G., Rehman S. (2020). The Balance between Work and Life for Subjective Well-Being: A Moderated Mediation Model. J. Open Innov. Technol. Mark. Complex..

[51] Peeters T., Van De Voorde K., Paauwe J. (2021). Exploring the Nature and Antecedents of Employee Energetic Well-Being at Work and Job Performance Profiles. Sustainability.

[52] Figueredo J.-M., García-Ael C., Gragnano A., Topa G. (2020). Well-Being at Work after Return to Work (RTW): A Systematic Review. Int. J. Environ. Res. Public Health.

[53] Buttler D. (2022). Employment Status and Well-Being Among Young Individuals. Why Do We Observe Cross-Country Differences?. Soc. Indic. Res..

[54] Pajic S., Buengeler C., Den Hartog D.N., Boer D. (2021). The moderating role of employee socio-economic status in the relationship between leadership and well-being: A meta-analysis and representative survey. J. Occup. Health Psychol..

